# Seed germination in relation to the invasiveness in spiny amaranth and edible amaranth in Xishuangbanna, SW China

**DOI:** 10.1371/journal.pone.0175948

**Published:** 2017-04-17

**Authors:** Juan Ye, Bin Wen

**Affiliations:** 1 Center for Integrative Conservation, Xishuangbanna Tropical Botanical Garden, Chinese Academy of Sciences, Mengla, Yunnan, China; 2 University of the Chinese Academy of Sciences, Beijing, China; University of Vigo, SPAIN

## Abstract

Both spiny and edible amaranths (*Amaranthus spinosus* and *A*. *tricolor*) are exotic annuals in China that produce numerous small seeds every year. Spiny amaranth has become a successful invader and a troublesome weed in Xishuangbanna, but edible amaranth has not, although it is widely grown as a vegetable there. As seed germination is one of the most important life-stages contributing to the ability of a plant to become invasive, we conducted experiments to compare the effects of high temperature and water stress on seed germination in two varieties each of spiny amaranth and edible amaranth. Overall, the seeds of both amaranth species exhibited adaptation to high temperature and water stress, including tolerance to ground temperatures of 70°C for air-dried seeds, which is consistent with their behavior in their native ranges in the tropics. As expected, the invasive spiny amaranth seeds exhibited higher tolerance to both continuous and daily periodic high-temperature treatment at 45°C, and to imbibition-desiccation treatment, compared to edible amaranth seeds. Unexpectedly, edible amaranth seeds exhibited higher germination at extreme temperatures (10°C, 15°C, and 40°C), and at lower water potential (below -0.6 MPa). It is likely that cultivation of edible amaranth has selected seed traits that include rapid germination and germination under stressful conditions, either of which, under natural conditions, may result in the death of most germinating edible amaranth seeds and prevent them from becoming invasive weeds in Xishuangbanna. This study suggests that rapid germination and high germination under stress conditions—excellent seed traits for crops and for many invasive species—might be a disadvantage under natural conditions if these traits are asynchronous with natural local conditions that support successful germination.

## Introduction

Biological invasion is a serious threat to native agriculture and natural ecosystems worldwide [1,2,3]. Taking Xishuangbanna in Yunnan as an example—one of the regions suffering most from invasive plant species in China—a preliminary study reported that there are 75 invasive plant species from 58 genera in 31 families, with some of them, such as Crofton weed, Mexican sunflower, and spiny amaranth, causing great harm to agricultural production and the local environment [4].

One of the most urgent issues in invasion biology is to reveal why some alien species became invasive while others do not [5,6]. For this purpose, comparative investigations of closely related species are frequently employed in biological invasion studies, such as those of Mandak [7], Cervera and Parra-Tabla [8], and Luo and Cardina [9]. In Xishuangbanna there is a pair of such species, the spiny and edible amaranths. Both are alien annual herbs, and spiny amaranth (*Amaranthus spinosus* L.) has developed into an invasive species in the region while edible amaranth (*Amaranthus tricolor* L.) has not. Spiny amaranth is native to tropical America, but now is considered an invasive plant worldwide [10,11]; in China, it is abundant in the tropical, subtropical, and warm temperate regions [12,13], with its first appearance documented in Macao in the 1930s [13]. It is a common invasive weed in Xishuangbanna [4,12], where it often grows in abandoned areas and cornfields. A prolific seed producer, its vast reproductive capacity and aggressive growth cause competition with crops for water and nutrition, which may result in crop failure [14,15]. Furthermore, its spines make control of this weed by manual methods very difficult [10]. Edible amaranth has a long introduction history and wide cultivation in China, but unlike the spiny amaranth, this species has not become a troublesome weed in Xishuangbanna although escaped plants can occasionally be found there. Hopefully, a comparative study of the spiny and edible amaranths will help to increase our knowledge of plant invasiveness.

In the plant life cycle, seed germination is an important development phase, playing a critical role in seedling establishment and overall environmental adaptation [16,17,18]. The ability of a species to germinate rapidly under a broad range of environmental conditions has been regarded as a critical trait for invasive species [19,20]. As both spiny and edible amaranths are annual herbs, with seed germination being their unique methods to establish seedlings in the course of their invasion, germination patterns must have important effects on their invasion success. We hypothesized that spiny amaranth has higher germinability under various conditions than edible amaranth, and that this contributes to its invasiveness.

In this study, we investigated the effects of temperature and water stress on seed germination in spiny and edible amaranths in order to improve our understanding of the noxious and invasive characteristics of spiny amaranth, and the contribution of seed germination to biological invasiveness. A sound understanding of the germination biology of spiny amaranth will also be useful for controlling the spread of this invasive species.

## Materials and methods

### Plants

Two varieties of spiny amaranth (*Amaranth**us spinosus* L.) can be found in Xishuangbanna, one with green stems and leaves and the other with red stems and leaves; in this paper we refer to these as green and red spiny amaranths, respectively. We found that green spiny amaranth is more common in fields in Xishuangbanna. Edible amaranth (*Amaranth**us tricolor* L.), widely cultivated as a vegetable in China, has many varieties produced by selective breeding. In this study, two varieties were chosen to match the characteristics of the spiny amaranth, with either green stems and leaves or red stems and leaves, and they are referred to here as green and red edible amaranths, respectively.

### Seed sources

Spiny amaranth seeds used in this study were harvested from populations growing on abandoned land or along the roadside in August 2015 in Menglun, where the Xishuangbanna Tropical Botanical Garden of the Chinese Academy of Sciences is located (21°55′ N, 101°15′ E). Seeds collected from randomly selected plants were pooled, cleaned, air-dried, and kept at 15°C prior to experimental usage. Seeds of edible amaranths were recently purchased in sealed containers from local commercial suppliers and also kept at 15°C.

After air-drying, the red and green spiny amaranth seeds used had 100-seed weights of 24.50 ± 0.32 mg and 22.30 ± 0.30 mg, respectively, and their moisture content was 16.03 ± 0.47% and 16.40 ± 0.26%, respectively. For red and green edible amaranths, these values were 68.72 ± 0.92 mg and 91.27 ± 0.71 mg, and 13.82 ± 0.04% and 13.99 ± 0.05%, respectively. Germination tests conducted before the experiments began found high seed viability (germination >90% for spiny amaranths, and >95% for edible amaranths).

### Experimental arrangements

In line with our previous study [21], we designed the following series of experiments to investigate the effects of high temperature and water stress, applied in different ways, on seed viability and germination.

#### High-temperature tolerance of quiescent seeds

To investigate the effects of extreme high temperature on seed viability, a temperature range from 30°C to 95°C with steps of 5°C was established using a water bath [21]. For each variety of each species, two triangular flasks containing about 350 seeds each were used at each testing temperature. We also tested the effects of seed hydration. The seeds in one flask were kept air-dried during heating while those in the other were permitted to imbibe a few drops of deionized water half an hour before heat treatment. After heating for half an hour at the indicated experimental temperature, the seeds were sown on 1% water agar for viability assessment.

#### Effects of incubation temperature and light on seed germination

Germination was determined by sowing seeds on 1% water agar in Petri dishes, and incubating them at temperatures from 10°C to 45°C in 5°C increments, and also at an alternating temperature of 18/28°C, with a 12-h photoperiod of 25 μmol m^-2^ s^-1^ irradiance provided by white fluorescent lamps. For the dark treatment, dishes were wrapped in a double layer of aluminum foil.

#### Effects of water availability on seed germination

Seeds were incubated in solutions with an incremental osmotic potential from 0 to -1.0 MPa (for spiny amaranths) or to -1.5 MPa (for edible amaranths), to investigate the effects of water stress on germination. The water potentials were created by either polyethylene glycol (PEG) 8000, following Michel [22], or NaCl, following Lang [23]. Seeds sown on filter paper discs moistened with testing solutions in Petri dishes were incubated at 30°C in light for germination. To minimize moisture loss during the experiment, the Petri dishes were sealed in resealable double-clear plastic bags. Seed germination was scored twice a week, with filter papers and testing solutions refreshed simultaneously. Six weeks later, the non-germinated seeds were transferred to filter papers moistened with deionized water to test if the seeds were still viable.

#### Effects of continuous heat treatment on seed germination

Since the above experiment found a high rate of germination when seeds were incubated at 40°C, a higher temperature of 45°C was used to determine the response to continuous and periodic heat stress treatment, an approach different from that used in previous studies [18,21]. Seeds were sown on water agar as described above, and placed in an incubator at 45°C. The heat stress treatment duration ranged from 12 h to 72 h in 12-h increments, and then to 240 h in 24-h increments. After heat stress treatment for a given period, the dishes containing the seeds were withdrawn from the 45°C incubator and placed in an incubator at 35°C to check seed viability.

#### Effects of periodic high temperature on seed germination

In this experiment, seeds sown on 1% agar were exposed to alternating temperatures of 45°C and 35°C for periods of 1/23 h, 2/22 h, 3/21 h, 4/20 h, 5/19 h, 7/17 h, 9/15 h, 12/12 h, 15/9 h, 18/6 h, and 21/3 h to determine the germination response to daily heat shocks of varying duration.

#### Desiccation interruption during seed germination

To investigate the effects of imbibition-dehydration treatment on seed germination, seeds were sown on filter paper discs moistened with deionized water in Petri dishes, incubated at 30°C, and then regularly sampled after incubation for different durations and air-dried at 50% RH and 15°C for 72 h, with an additional germination scoring just before drying. After this interruption, they were watered and incubated at 30°C for viability assessment. Seed germination was scored twice a week, with deionized water replenished simultaneously when necessary.

### Seed viability and germination assessment

In this study, 50 seeds × 6 replicates per variety per species were used for each treatment. Unless stated otherwise, the seeds were scored once a week for at least six weeks. Each seed was considered to have germinated, or survived the stress experiments, when a visible radicle could be discerned; each seed was removed when a cotyledon was grown (seedling). Non-germinated seeds were subjected to a simple pressure test to assess viability: white, firm embryos were considered as viable, and brown, soft embryos were nonviable [24].

### Data analysis

Seedling and germination or survival percentages were separately taken as dependent variables for data analysis after arcsine-square-root-transform. A two- or three-way analysis of variance (ANOVA) was employed, treating species and all applied environmental factors as fixed effects. Instead of testing variety effect, we simply treated it as a random effect because both spiny amaranth varieties and neither edible amaranth ones are invasive in Xishuangbanna. This method allowed testing the species effect as a whole. In water availability experiment, only osmotic potential levels shared by both species were included in statistical analysis; while germination data at constant temperature of 35°C was included as a control in analysis of effect of continuous and periodical heating treatment. All analyses were carried out using SPSS 16.0 for Windows. Data are presented as means and standard errors.

## Results

The germination results of the amaranth seeds were significantly affected by all environments tested (Table 1). Most of the species effects as well as all the species-by-environment interactions were significant. These results indicate a complicated germination response by the species and varieties exposed to various environmental conditions.

**Table 1:** Analysis of variance for seedling and germination or survival percentage of amaranths seeds.

Experiment	Effect	df	*F*-value		
			Seedling	Germination	Survival
High-temperature tolerance	Species	1	106.160***		187.784***
	Hydration status	1	1246.207***		1854.386***
	Temperature	13	917.843***		833.094***
	S×H	1	11.734***		37.195***
	S×T	13	6.610***		4.338***
	H×T	13	173.710***		206.197***
	S×H×T	13	4.605***		5.029***
Incubation temperature and Illumination^1^	Species	1	1057***	1348***	
	Illumination	1	36.637***	22.001***	
	Temperature	7	689.347***	947.200***	
	S×I	1	5.652* (*p* = 0.018)	15.992**	
	S×T	7	151.966***	173.748***	
	I×T	7	22.564***	29.974***	
	S×I×T	7	16.450***	9.588***	
Water availability^2^	Species	1	2044.679***	2196.732***	
	Reagent	1	0.305 (*p* = 0.581)	1.31 (p = 0.253)	
	Water potential	8	265.858***	254.367***	
	S×R	1	111.262***	120.225***	
	S×W	8	83.441***	80.084***	
	R×W	8	23.981***	23.563***	
	S×R×W	8	9.328***	8.564***	
Continuous heat treatment^3^	Species	1	56.924***		72.233***
	Heating duration	13	307.538***		174.422***
	S×H	13	14.274***		15.782***
Periodic heat treatment^3^	Species	1	68.996***	1.944(*p* = 0.164)	
	Heating duration	11	226.960***	41.250***	
	S×H	11	33.767***	33.099***	
Desiccation interruption	Species	1	3.759(*p* = 0.0535)		7.999 **
	Imbibition time	11	149.432***		150.007***
	S×I	11	23.05***		24.986***

"*" *p*<0.05;

"**" *p*<0.01;

"***" *p*<0.001

^1^The alternative temperature of 18/28°C was not included in this analysis;

^2^Only osmotic potential levels shared by both species were included in this analysis;

^3^Germination data at constant temperature of 35°C was included in these analysis as control (0 h at 45°C).

### High-temperature tolerance of quiescent seeds

**Fig 1:**
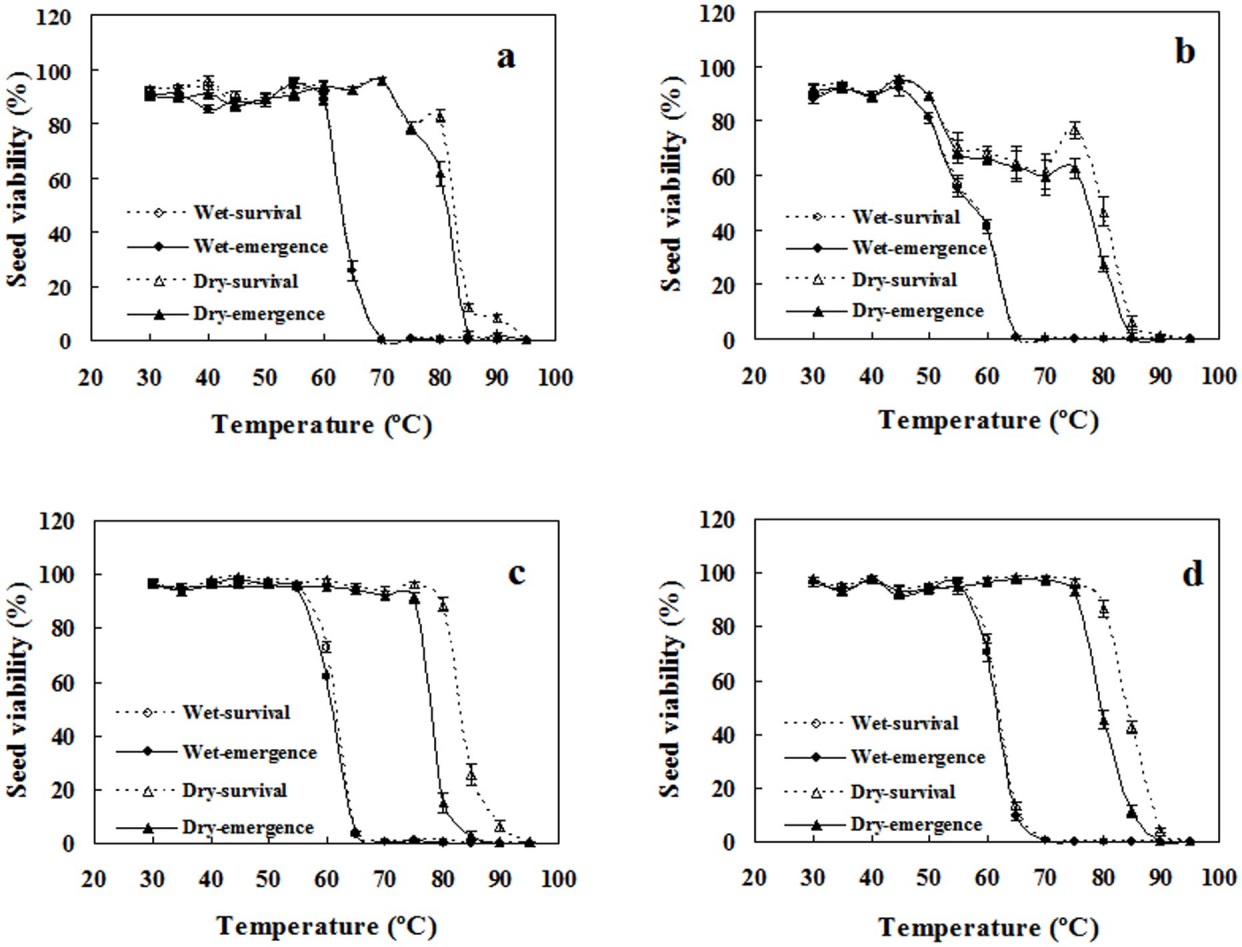
Effects 30-min heat shocks from 30°C to 95°C on viability of air-dried (dry) and imbibed (wet) seeds. **a**. red spiny amaranth; **b**. green spiny amaranth; **c**. red edible amaranth; **d**. green edible amaranth. Survival and seedling percentages are expressed as means ± SE of six replicates of 50 seeds.

The three-way ANOVA indicated that species, seed hydration, testing temperature, and their interaction all had significant effects on seed viability, assessed by both survival and seedling percentage (Table 1). The responses to high temperature were characterized by two common features. First, all germination reduction occurred in a narrow temperature range, with its low side varying from 55°C to 65°C and its high side from 85°C to 90°C. Below this range, nearly all seeds germinated, while above it, no seeds survived. Second, as shown by the discrepancy between the viability curves for seeds with different hydration, air-dried seeds showed markedly higher tolerance of short-term high temperature than did imbibed seeds (Fig 1). Furthermore, only air-dried seeds exhibited marked differences between seedling and survival percentage after heating above 70°C (Fig 1).

Red spiny amaranth seeds showed greater tolerance of high temperature than any other seeds used. For example, a 30-min treatment at 60°C did not impair viability of red spiny amaranth imbibed seeds, but reduced the viability of edible amaranth imbibed seeds to 60–70%; after heating at 80°C, their air-dried seed germination percentage was 60% for red spiny amaranth, 30% for green spiny amaranth, and 15% and 40% for red and green edible amaranths, respectively (Fig 1).

### Effects of incubation temperature and light on seed germination

**Fig 2:**
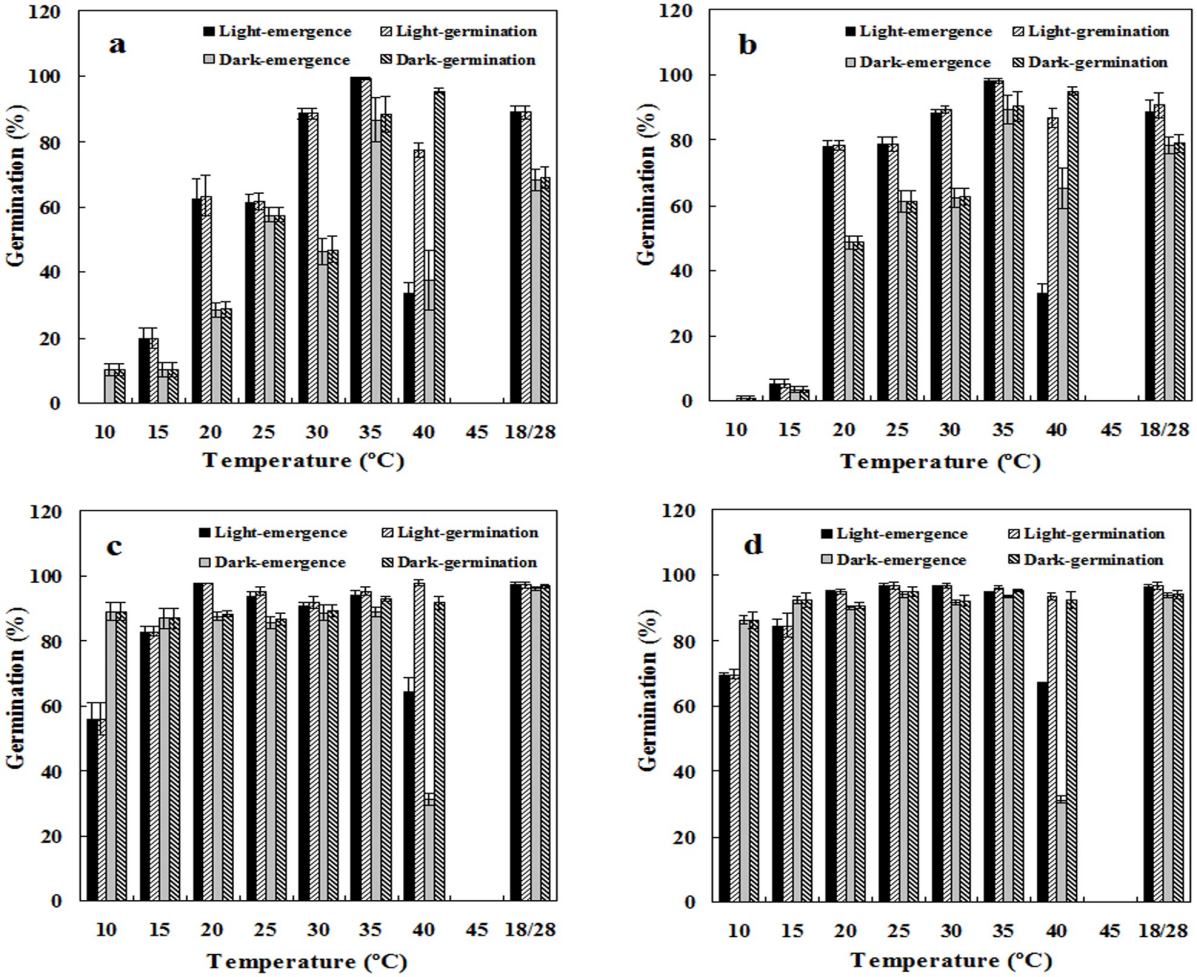
Effects of incubation temperatures and light on seed germination. **a**. red spiny amaranth; **b**. green spiny amaranth; **c**. red edible amaranth; **d**. green edible amaranth. Seeds were incubated at a constant temperature from 10°C to 45°C, and at 18/28°C, with periodic illumination (**light**) or under full dark (**dark**). Germination and seedling percentages are expressed as means ± SE of six replicates of 50 seeds.

Species, temperature, light, and their interactions all significantly affected seed germination (three-way ANOVA; *p* < 0.05 for both seedling and germination percentage; Table 1). Evidently, edible amaranths had better germination performance than spiny amaranths in diverse conditions, especially at the lower temperatures of 10°C and 15°C. Although light was not a requisite for germination, seeds in light germinated better than those in dark, except those incubated at 10°C and 40°C; furthermore, in the range from 20°C to 30°C, light improved germination much more in spiny amaranths than in edible amaranths. Germination and seedling percentage in spiny amaranths increased as incubation temperature increased from 15°C to 30°C, peaked at 35°C, and then decreased markedly at 40°C. In comparison, incubation temperatures from 20°C to 35°C did not influence edible amaranth germination. Although both species still had ≥80% germination at 40°C, only approximately half of the germinated seeds formed morphologically normal seedlings. At 45°C no germination happened and all seeds molded after approximately 10 days of incubation (Fig 2). Fluctuating temperature is not a requisite treatment for germination, although seeds incubated at 18/28°C had high germination percentages of 70–100%.

### Effects of water availability on seed germination

**Fig 3:**
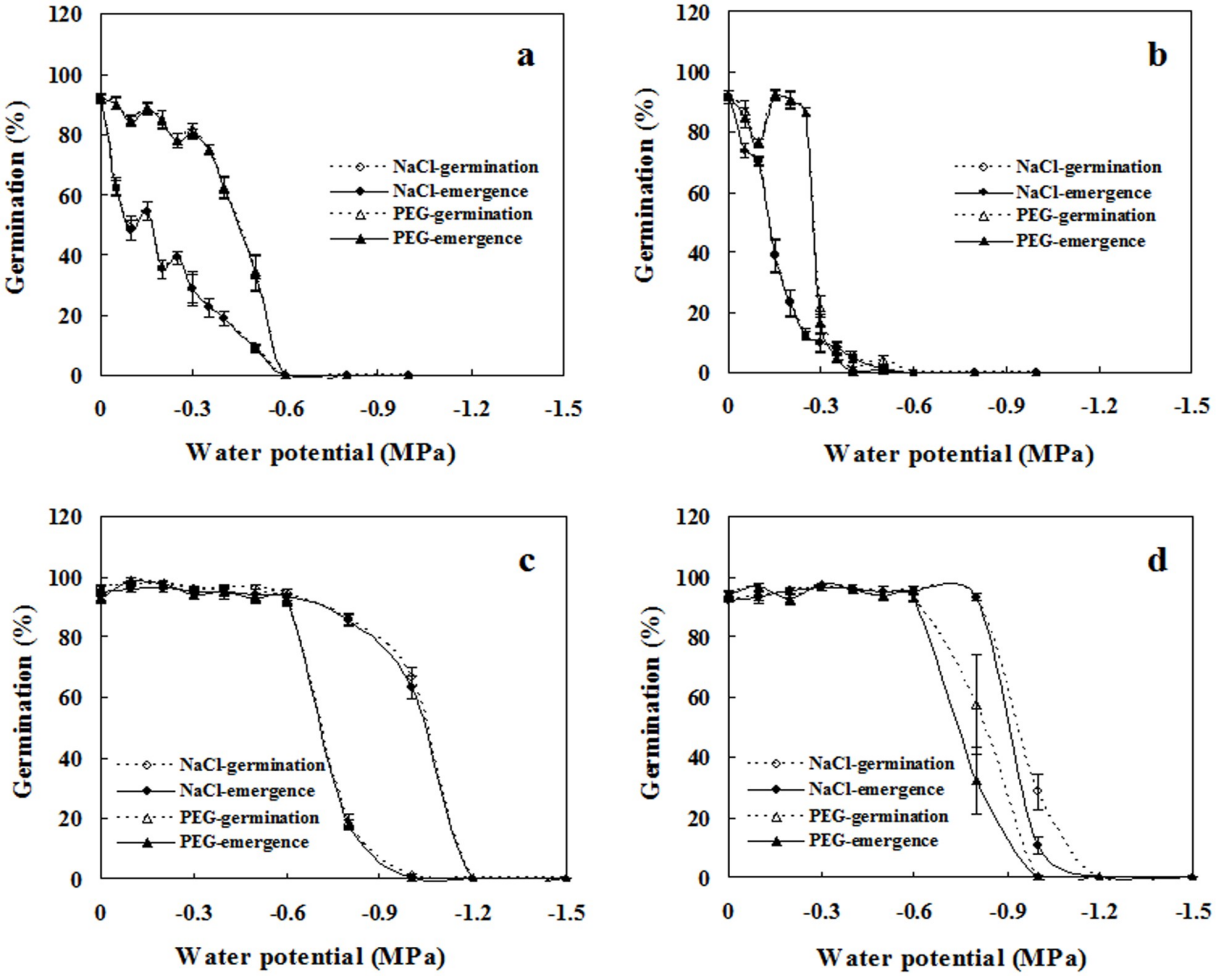
Effects of water potential on seed germination. **a**. red spiny amaranth; **b**. green spiny amaranth; **c**. red edible amaranth; **d**. green edible amaranth. Water potentials were created by **PEG** 8000 and **NaCl**. Germination and seedling percentages are expressed as means ± SE of six replicates of 50 seeds.

Great differences in seed germination in response to water stress were found between spiny and edible amaranths (three-way ANOVA; *p* < 0.001 for species, water potential, and their interaction, including that with reagent; Table 1). As water stress increased, germination in spiny amaranth seeds was gradually inhibited, with no germination occurring under -0.6 MPa. In contrast, edible amaranth seeds exhibited no germination decrease at all at water stress levels of -0.6 MPa; after -0.6 MPa, the germination percentage of edible amaranth seeds decreased progressively, with complete inhibition at -1.2 MPa. Water stress created by PEG and NaCl affected germination differently in the two species. Between -0.05 MPa and -0.5 MPa, seeds of spiny amaranths treated with NaCl solutions had lower germination than those treated with PEG solutions under equal water potentials, indicating ionic toxic effects of NaCl. However, at water potentials between -0.6 and -1.2 MPa, edible amaranth was more sensitive to PEG. Seedling percentages significantly differed from germination percentages only in green edible amaranth seeds incubated at -0.8 and -1.0 MPa (Fig 3). When non-germinated seeds, after a 6-week stress treatment, were removed from the testing solutions and incubated in deionized water, most of them germinated.

### Effects of continuous heat treatment on seed germination

**Fig 4:**
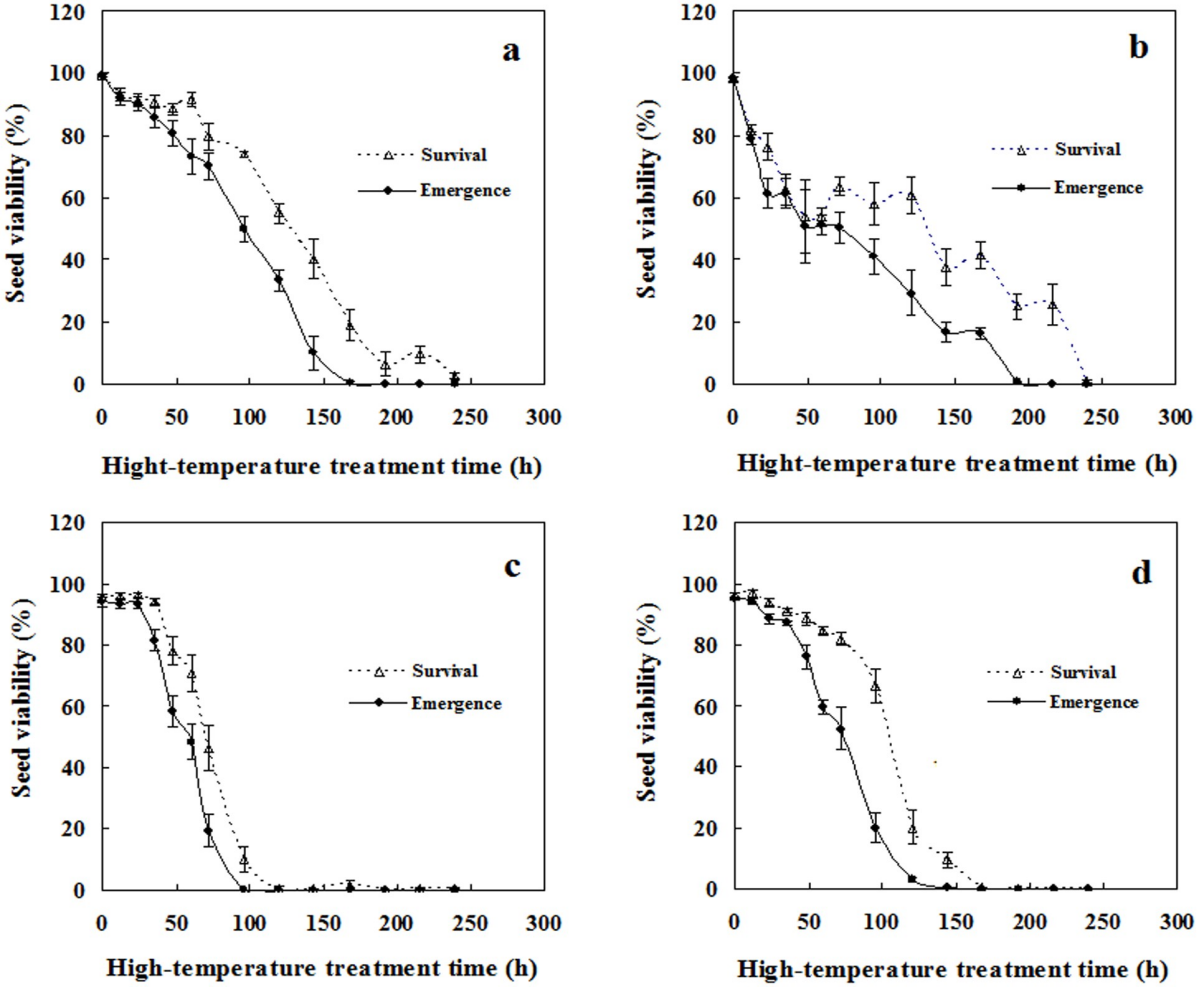
Effects of continuous high-temperature stress on seed viability. **a**. red spiny amaranth; **b**. green spiny amaranth; **c**. red edible amaranth; **d**. green edible amaranth. Seeds were subjected to heat shock at 45°C, and incubated at 35°C after they were released from stress. Survival and seedling percentages are expressed as means ± SE of six replicates of 50 seeds.

Spiny and edible amaranth seeds had obviously different tolerances to a continuous high temperature of 45°C (two-way ANOVA; *p* < 0.001 for species, heating time, and their interaction). As treatment duration increased, both survival and seedling percentage linearly decreased, although the curves for edible amaranth seeds had very narrow shoulders (within 24 h, Fig 4c and 4d). Obviously, edible amaranth seeds exhibited higher sensitivity to continuous high temperature than spiny amaranth seeds, for they lost viability more rapidly as heating time increased, and thus had lower germination after longer heat treatments. For example, after heat treatment for 96 h, seedling percentage was 50% and 40% for red and green spiny amaranths, but 0% and 20% for red and green edible amaranths, respectively. In this experiment, germination percentage was obviously greater than seedling percentage for most treatments, suggesting that many germinated seeds failed to form seedlings (Fig 4).

### Effects of periodic high temperature on seed germination

**Fig 5:**
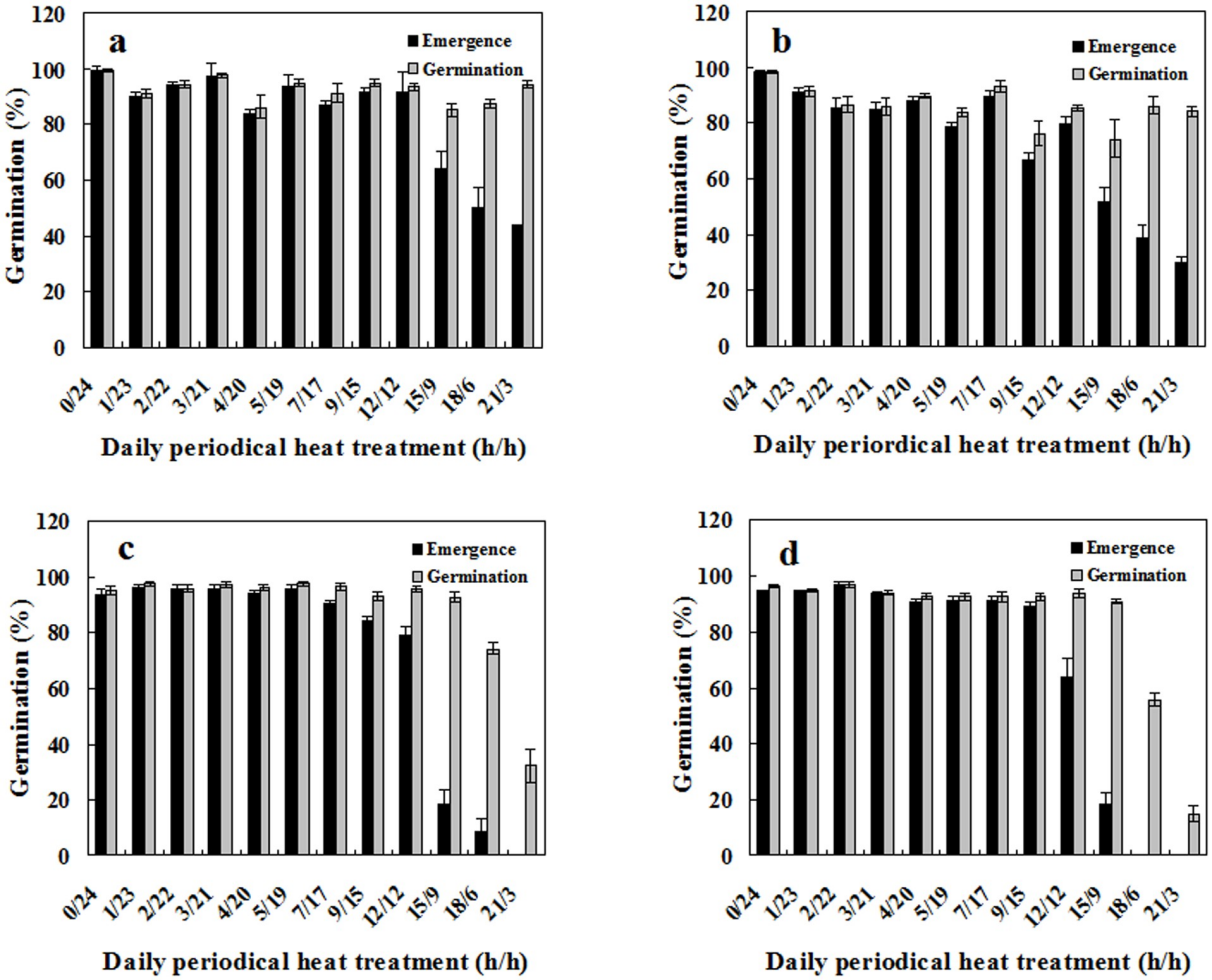
Effects of daily periodic high-temperature stress on seed germination. **a**. red spiny amaranth; **b**. green spiny amaranth; **c**. red edible amaranth; **d**. green edible amaranth. Seeds were subjected to 45°C and 35°C (h/h) alternately. Germination and seedling percentage are expressed as means ± SE of six replicates of 50 seeds.

Both species and daily heating duration had a significant influence on germination (two-way ANVOA; *p* < 0.001 for heating duration and its interaction with species, but only for species assessed by seedling percentage; Table 1). Edible amaranth seeds responded only to a daily heating period above 7 h; below this threshold, 90% of seeds germinated and formed morphologically normal seedlings. Spiny amaranth seeds also responded to short heating periods, but with fluctuating effects (Fig 5). Substantial germination reduction occurred in both species after the daily heating period exceed 12 h, with seedling percentage decreasing first with the increase of the heating period, and then germination percentage decreasing. Furthermore, the difference between seedling and germination percentage increased. When the daily heating period reached 18–21 h, only a few edible amaranth seedlings formed even though many seeds germinated. In comparison, spiny amaranth seeds retained a seedling percentage of 30–50%, suggesting higher tolerance to this stress in spiny amaranth seeds (Fig 5).

### Effects of desiccation interruption during seed germination

**Fig 6:**
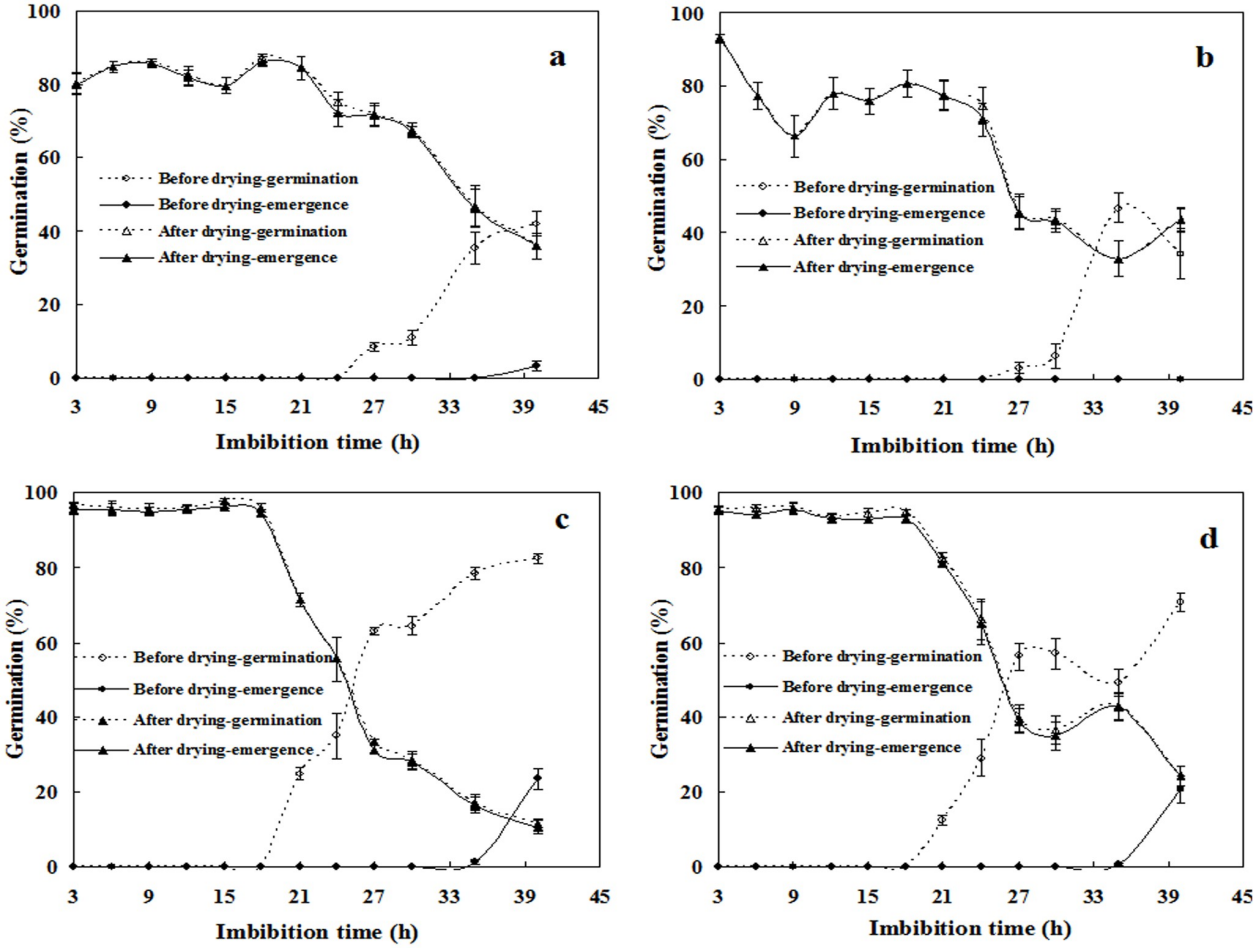
Effects of dehydration after imbibition on seed germination. **a**. red spiny amaranth; **b**. green spiny amaranth; **c**. red edible amaranth; **d**. green edible amaranth. After imbibition for the indicated period, seeds were dried for 72 h under 50% RH at 15°C, and then germinated for viability assessment. Survival and seedling percentages, expressed as means ± SE of six replicates of 50 seeds, were used to score germination before and after drying treatment, separately.

Imbibition duration before desiccation interruption had a significant influence on the viability of seeds experiencing an imbibition-desiccation cycle. Furthermore, spiny amaranths differed from edible amaranths significantly in this respect, but only when assessed by survival percentage (two-way ANOVA; *p* < 0.01 for imbibition duration and its interaction with species; Table 1). For seeds of both red and green edible amaranths, up to 21 h of imbibition prior to desiccation was harmless, but seedling and survival percentage decreased abruptly after this turning point. For green and red spiny amaranth seeds, this turning point occurred around 24 h and 30 h, respectively; their seedling and survival percentage fluctuated before this point, and then decreased sharply. Nevertheless, spiny amaranth seeds had higher tolerance to desiccation interruption than did edible amaranth seeds, especially when the imbibition duration exceeded 21 h. For example, desiccation after 21 h of imbibition reduced the viability of red and green spiny amaranth seeds to 85% and 80%, respectively (Fig 6a and 6b), and that of red and green edible amaranth seeds to 70% and 80%, respectively (Fig 6c and 6d). However, after 40 h of imbibition, these values decreased to 36%, 40%, 10%, and 24%, respectively (Fig 6). This may be related to their germination rate, for edible amaranth seeds had faster germination than spiny amaranths: there were 42% red and 35% green spiny amaranth seeds germinated after 40 h of imbibition, just before desiccation treatment, while these values were 82% and 70% for red and green edible amaranth seeds, respectively (Fig 6).

## Discussion

This study investigated the germination requirements and seed germination of two congeneric species, spiny and edible amaranths, under high-temperature and water stress. Seeds of both species germinated across a wide temperature range, from 10°C to 40°C, with their maximum germination occurring at 35°C, and they exhibited strong tolerance to extreme high temperature of 70°C in air-dried seeds, and to continuous and daily periodic heat treatment at 45°C. All these characteristics indicate their adaptation to high-temperature conditions in the tropics, a seed trait that has been rarely reported even in tropical seeds such as *Brunnichia ovata* [25] and *Trianthema pottulacastrum* [26].

Some species in the genus *Amaranthus* have become troublesome weedy pests [27,28] and successful invaders [29] when introduced outside of their natural range, including redroot pigweed (*Amaranthus retroflexus*) [30], slender amaranth (*A*. *viridis*) [31], Palmer amaranth (*A*. *palmeri*) [27], and common waterhemp (*A*. *rudis*) [27]. Spiny and edible amaranths share many common traits: both are short-lived exotic herbs in China, they produce large numbers of small seeds each year, and they shed seeds in late summer to autumn. Furthermore, the tender stems and leaves of spiny amaranths are collected in the wild for use by local people. We were interested in understanding why one of them developed into a noxious weed in Xishuangbanna while the other failed to do so. Because species with great phenotypic plasticity and environmental tolerance have high tolerance to numerous environments and may easily find suitable habitats in the introduced localities, Ren and Zhang argued that both phenotypic plasticity of environmental tolerance and evolutionary adaptation to physical environment are important mechanisms of biological invasion [32]. This is exhibited in seed germination as invasive species have prolific and rapid germination in the presence of a wide range of environmental cues compared to native species. The seed traits of prolific and rapid germination may be a successful strategy for facilitating invasion because they permit quick responses to increased resource availability on the one hand [33], and on the other hand allow early seedling emergence and establishment, providing a head start in growth and the advantage of occupying space to the exclusion of neighbouring plants [34]. The contribution of prolific and rapid seed germination to invasiveness has been proved by numerous previous studies, including those of Cervera and Parra-Tabla [8], Pyšek and Richardson [16], Wainwright and Cleland [33], and Radford and Cousens [35]. A recent example came from Bochenek et al. [36] who reported that invasive *Solidago gigantea* seeds maintained high seed vigor after being stored under a wide range of temperatures in both dry and moist conditions, and stratification and after-ripening caused only minor reductions in germination rate and final germination percentage. Erfmeier and Bruelheide [34] even found that invasive *Rhododendron ponticum* populations exhibited significantly higher germination rates than native ones. Our study compared seed tolerance and stress germination in two amaranths, and found that the invasive spiny amaranths demonstrated higher tolerance to continuous and daily periodic high temperature and to imbibition-desiccation treatment than did the seeds of the non-invasive edible amaranth. This was consistent with the findings of our previous reports on the invasive bamboo piper [21] and Mexico sunflower [18], and provides insights into the mechanisms that allow spiny amaranth to be a successful invader.

Contrary to our hypothesis, however, we found that the non-invasive edible amaranth seeds germinated across wider temperature ranges and water conditions than spiny amaranth seeds. In fact, the germination performance of spiny amaranth seeds was comparable to that of edible amaranth only at 35°C; their differences were distinct, especially at 10°C, 15°C, and 40°C, with light germination occurring only to edible amaranths at 10°C. Comparison of germination under water stress yielded clear results. Spiny amaranth was more sensitive to water restriction, with seed germination completely inhibited when water potential was reduced to -0.6 MPa, in agreement with a previous report [10]. In contrast, germination inhibition of edible amaranth seeds occurred between -0.8 and -1.2 MPa; water stress of -0.6 MPa did not reduce its germination at all. Prolific and rapid germination is therefore not necessarily associated with invasion success. Similar results have been reported previously by Luo and Cardina [9], Mandák [7], and Milbau et al. [37].

Given the combination of germination traits and seed-maturation traits of these species, we assumed that the soil conditions in Xishuangbanna would lead to futile germination of edible amaranth seeds, i.e., death of germinated seeds soon after germination. Futile germination would prevent this species from establishing a reserve of viable seeds in the soil seed bank, an important life-history stage for most weedy and invasive species [29]. This assumption was supported by our unpublished field experiments in which spiny and edible amaranth seeds, both red and green varieties, were buried in two habitats, one group in bare fields and the other in rainforest, and were regularly exhumed for viability assessment. Spiny amaranth seeds remained quiescent and viable for at least 10 months in the fields, while almost all edible amaranth seeds germinated in the soil within 3 weeks after burial (experiments ongoing and data unpublished).

As discussed above, the seed traits of higher and more rapid germination across a wide range of environments have been successfully associated with plant invasiveness in many invasive species, but they led to futile germination in edible amaranth seeds in Xishuangbanna. This indicates an asynchrony between seed germination in edible amaranth seeds and the habitat conditions at this locality. We recognize that the edible amaranths used in this study were cultivars of this species, so their germination traits under temperature stress and low water potential were acquired through artificial, and not natural selection. Cultivation has selected edible amaranth seeds for rapid germination and germination under stress conditions, but has failed to synchronize seed germination with conditions favorable for species survival. Thus, the trait of higher and more rapid germination under stress conditions can be either an advantage or a disadvantage, depending on whether these traits are adaptive in local conditions. We do not exclude the possibility that edible amaranths could become successful invaders in suitable conditions, but in Xishuangbanna the asynchrony in this species between seed germination and habitat conditions must be a key limiting factor that affects its survival in the wild. Adaptation to local environmental conditions is thus a context-specific process, and successful invasive species must not only exhibit high tolerance but must also successfully track seasonality, synchronizing successful seedling recruitment with favorable habitat conditions [38].
